# Embolic Stroke Diagnosed by Elevated D-Dimer in a Patient With Negative TEE for Cardioembolic Source

**DOI:** 10.1177/2324709614560907

**Published:** 2014-12-10

**Authors:** Irina Y. Sazonova, Roja Pondicherry-Harish, Nikhil Kadle, Gyanendra K. Sharma, Ramon E. Figueroa, Vincent J. B. Robinson

**Affiliations:** 1 Georgia Regents University, Augusta, GA, USA

**Keywords:** transesophageal echocardiography, atrial thrombosis, stroke, D-dimer

## Abstract

We report a case of cerebrovascular accident with thromboembolic stroke etiology in a patient who had atrial flutter and negative transesophageal echocardiography (TEE) results. The increased D-dimer levels (1877 ng/mL) initiated referral for magnetic resonance imaging and magnetic resonance angiography of the brain that showed classic recanalization of an embolic thrombus in the angular branch of the left middle cerebral distribution. The D-dimer level of this patient was normalized after 3 months of anticoagulation therapy. Although TEE is considered the gold standard for evaluation of cardiac source of embolism, exclusion of intracardiac thrombus with TEE alone does not eliminate the risk of thromboembolic events. This case highlights the utility of D-dimer as a potential adjunct in the decision-making process to guide investigation of thromboembolism, determine subsequent therapy, and hence reduce the risk of embolic stroke recurrence.

## Introduction

Despite a decade of intense public education and medical advancement, stroke continues to represent a leading cause of long-term disability and death.^1^ The cost burden associated with ischemic stroke is also substantial compared with other diseases.^2,3^ According to multiple clinical studies, the cardioembolic subtype of ischemic event has the most unfavorable prognosis, being associated with an increased risk of death or stroke recurrence, less favorable clinical outcomes including the risk of bleeding, and therapeutic benefits compared with other subtypes.^4-6^ Thus, it is critical for ischemic stroke patients to be rapidly evaluated and considered for possible cardioembolic sources to guide therapy as well as to reduce the physical and financial burden of poststroke management.^7,8^ Currently, transesophageal echocardiography (TEE) is the gold standard to diagnose cardiac thrombi in stroke and transient ischemic attack patients with undetermined cause.^9,10^ We present an evidence-based case of cerebral embolism when TEE did not show any cardiac source thrombus. Further investigation for cardioembolic stroke was initiated due to a positive risk marker, the increased plasma D-dimer level.

## Case Report

**Figure 1.**
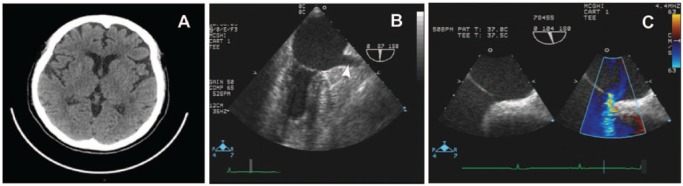
(A) CT head without contrast found no evidence of acute intracranial process such as thrombus, hemorrhage, mass effect, or hydrocephalus. (B, C) TEE images showed no evidence of thrombus in the left atrial appendage nor valvular vegetations. It showed a normal left ventricular function with ejection fraction of greater than 55% and secondum ASD (8.2 mm size with qp:qs ratio of 1.6:1).

A 79-year-old female presented for routine follow-up in our cardiology clinic. She had a 20-year history of an ostium secundum atrial septal defect (ASD) for which she refused closure and had a prior cerebrovascular accident without sequel. The patient also had a history of hypertension and dyslipidemia. The patient reported having an episode of right upper limb weakness 6 days previously. The symptoms lasted for 20 minutes before completely resolving. On examination, her heart rate was 52/min, blood pressure 118/60 mm Hg, and the chest was clear to auscultation. The glucose level was 80 mg/dL. The patient did have a hypercoagulability workup as follows: protein C activity (113%), protein S activity (124%), fibrinogen (313 mg/dL), ATIII (85%), APC ratio (3.0). Cardiac exam revealed fixed splitting of the second heart sound. Her neurological exam was normal. A 12-lead electrocardiogram (EKG) showed minor anterolateral T-wave changes, less pronounced than on previous EKG 6 months ago. There was no history or echocardiographic findings to suspect valvular vegetation. Initially the patient was diagnosed with a transient ischemic attack. The computed tomography (CT) of the head without contrast found no evidence of an acute intracranial process (Figure 1A). CT angiography of chest/pelvis/abdomen found no evidence of pulmonary embolus. A TEE was performed and showed no evidence of thrombus in the heart including the left atrial appendage (Figure 1B), no valvular vegetations, normal left ventricular function, and a small secondum ASD (Figure 1C).

**Figure 2.**
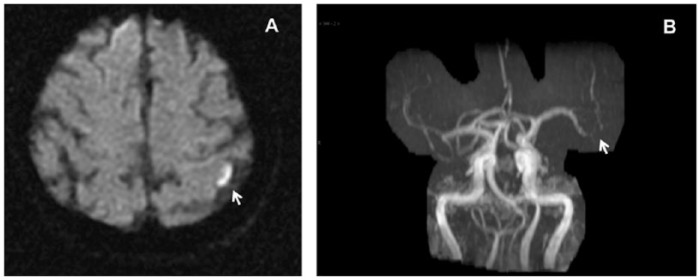
(A) Brain MRI showed a small acute left motor cortex infarct with spotty subcortical white matter ischemic lesions. The lesion in the left precentral gyrus correlated with the history of right upper extremity weakness. (B) MRA suggested the presence of an embolic stroke by identifying irregular segmental narrowing of the angular branch of the left middle cerebral artery, of concern for recanalization stenosis with either spontaneous fragmentation or lysed thrombus fragmentation.

The patient had impaired left atrial appendage function (LAAV = 39 cm/s), which is a potential risk for thromboembolism.^11^ The carotid duplex examination showed minimal plaque at both bifurcations but was otherwise unremarkable. Her D-dimer level was elevated with a critical value of 1877 ng/mL (reference range <250 ng/mL). A venous Doppler of lower extremities revealed no evidence of deep venous thrombosis, and likelihood of deep vein thrombosis was hence significantly reduced. In view of the high thromboembolic risk (significantly elevated D-dimer level and impaired LAAV), the patient was referred to magnetic resonance imaging (MRI) and magnetic resonance angiography (MRA) of her head. The MRI showed a small acute left motor cortex infarct with spotty subcortical white matter ischemic lesions suggesting hypoperfusion injury or small embolic lesion with rapid fragmentation of thrombus, consistent with the patient’s presentation (Figure 2A). The MRA further suggested the presence of thromboembolism by identifying irregular segmental narrowing of the angular branch of the left middle cerebral artery (MCA) consistent with thrombus recanalization with either spontaneous fragmentation or thrombolyzed thrombus fragmentation (Figure 2B). Based on MRA findings and positive D-dimer level, the patient was reclassified with cardioembolic stroke, most likely paradoxical emboli via her ASD. As a result of the high cardioembolic risk and CHADS 3 status, the patient was started on Dabigatran therapy. Event monitoring for 30 days was requested by the neurology consultant. After percutaneous closure of her atrial septal defect, the event monitor results were obtained, showing no pacemaker requiring bradycardia with heart rates in excess of low 40s. However, she did have episodes of atrial flutter with controlled ventricular response and hence had to be continued on oral anticoagulation. After 3 months of anticoagulant therapy, the level of her plasma D-dimer was normalized to 218 ng/mL. Thus, the elevated D-dimer level and the impaired LAAV expedited the diagnosis of cardioembolic etiology of stroke and the implementation of appropriate management.

## Discussion

Lack of conclusive findings to suggest cardioembolic source on negative TEE, hematology tests, or other routine diagnostics generally results in delaying anticoagulation therapy or stopping further workup. Although a negative TEE obviates the need for prolonged anticoagulation prior to cardioversion, it does not eliminate the presence of intracardiac thrombi that may generate stroke in about 1% of patients.^10,12^ Considering a 15% risk of cardioembolic stroke recurrence, this case underlines the diagnostic challenge in a cost- and time-benefit algorithm of stroke classification. Prothrombotic markers, including D-dimer, were suggested to assess patients at high risk of thrombogenesis.^13,14^ Although D-dimer is widely used for pulmonary and deep vein thrombosis,^15-18^ it is still not applied in the routine practice for diagnosis of cardiac thrombi. Recent literature suggests D-dimer levels are significantly higher in the cardioembolic group of ischemic stroke than in the atherothrombotic and lacunar groups^19,20^ and are related to infarction volume and functional outcomes.^21^ D-dimer has also has been proposed for prediction of subsequent thromboembolic cardiovascular events in atrial fibrillation patients during oral anticoagulant therapy.^14,22,23^ However, the stratification value of routine D-dimer for acute cardioembolic management remains unclear. Our case describes an emerging role of the cost-effective D-dimer test in combination with cardiac morphological data in evaluating the cardioembolic etiology of stroke. This presents a unique example of how thromboembolic risk may be underestimated in patients who undergo TEE with negative results^10^ and how to develop further investigations, treatment, and prevention strategies. Our patient suffered recent neurologic impairment, but TEE showed no evidence of thrombus or vegetation, so a cardiac source of emboli could not be strongly imputed. However, MRA did show irregular segmental narrowing of the left MCA with recanalization, which may be consistent with an embolic-type minor stroke pattern rather than an in situ atherothrombotic event. An isolated lesion in a distal segment of the MCA is unlikely to be atheromatous in the absence of similar disease in more proximal segments. Vasospasm also would be unlikely in the patient clinical setting, especially in the absence of subarachnoid hemorrhage or meningitis (two of the most frequent triggers of vasospasm). The most plausible interpretation is, thus, an embolic lesion with thrombus fragmentation, which explains all the findings. The D-dimer test was crucial in pointing us toward the diagnosis of thromboembolic stroke likely secondary to paradoxical embolism, and allowing the patient to undergo the necessary preventive measures, including the closure of the ASD and anticoagulation therapy. It is also important to note that the patient did not have evidence of deep vein thrombosis, as a potential source of the increased D-dimer level. Therefore, it is possible that the cardioembolic subtype of stroke (due to a small cardiac thrombus or already embolized thrombus) is more common than has been previously recognized because of the difficulty of making this complex diagnosis. More clinical experience with D-dimer is needed, in order to reevaluate patients with high risk of cardioembolism and guide further investigations as become necessary. Such an approach could prevent future stroke recurrence, thus decreasing morbidity and associated health care cost. Thus, this case highlights the value of D-dimer and reviewing LAAV data when the TEE is negative to support the additional workup for the diagnosis of stroke etiology.
